# Procedure-Specific Risks of Robotic Simultaneous Resection of Colorectal Cancer and Synchronous Liver Metastases

**DOI:** 10.21203/rs.3.rs-2920026/v1

**Published:** 2023-05-16

**Authors:** Shannon N. Radomski, Sophia Y. Chen, Miloslawa Stem, Joy Zhou Done, Chady Atallah, Bashar Safar, Jonathan E. Efron, Alodia Gabre-Kidan

**Affiliations:** The Johns Hopkins University School of Medicine; The Johns Hopkins University School of Medicine; The Johns Hopkins University School of Medicine; The Johns Hopkins University School of Medicine; NYU Langone Health; NYU Langone Health; The Johns Hopkins University School of Medicine; The Johns Hopkins University School of Medicine

**Keywords:** robotic surgery, colorectal liver metastases, simultaneous resections, hepatic resection

## Abstract

An estimated 25% of patients with colorectal cancer (CRC) present with distant metastases at the time of diagnosis, the most common site being the liver. Controversy exists regarding the safety of a simultaneous versus staged approach to resections in these patients, but reports have shown that minimally invasive surgery (MIS) approaches can mitigate morbidity. This is the first study utilizing a large national database to investigate colorectal and hepatic procedure-specific risks in robotic simultaneous resections for CRC and colorectal liver metastases (CRLM). Utilizing the ACS-NSQIP targeted colectomy, proctectomy, and hepatectomy files, 1,550 patients were identified who underwent simultaneous resections of CRC and CRLM from 2016–2020. Of these patients, 311 (20%) underwent resections by an MIS approach, defined as an either laparoscopic (n = 241, 78%) or robotic (n = 70, 23%). Patients who underwent robotic resections had lower rates of ileus compared to those who had an open surgery. The robotic group had similar rates of 30-day anastomotic leak, bile leak, hepatic failure, and post operative invasive hepatic procedures compared to both the open and laparoscopic groups. The rate of conversion to open was significantly lower for robotic compared to laparoscopic group (9% vs. 22%, p = 0.012). This report is the largest study to date of robotic simultaneous CRC and CRLM resections reported in the literature and supports the safety and potential benefits of this approach.

## INTRODUCTON

Approximately 20–25% of patients with colorectal cancer (CRC) present with metastatic disease at the time of diagnosis [1]. For patients with resectable colorectal liver metastases (CRLM), surgical resection is the preferred treatment modality with a 5-year overall survival (OS) rate of 38% and median OS of 3.6 years [2]. Resection can be performed in a simultaneous or staged approach, although controversy exists in the literature regarding the safety of a simultaneous resection [3–5]. Recent small case reports and institutional series have reported on the benefits of minimally invasive liver surgery (MILS) in simultaneous resections, including decreased length of stay (LOS), lower blood loss, and decreased overall hospital costs compared to an open approach [4, 6]. The aim of this study was to report on procedure-specific outcomes of robotic simultaneous CRC and CRLM resections from a national database.

## METHODS

Adult patients with CRC who underwent simultaneous colorectal and liver resections between 2016 and 2020 were identified from the colectomy, proctectomy, and hepatectomy-targeted American College of Surgeons (ACS) National Surgical Quality Improvement Program (NSQIP) files. Further stratifications were made based on operative approach (laparoscopic, robotic, or planned open) and risk of procedure. Consistent with prior risk stratifications reported in the literature patients were divided into high-risk or low-risk groups based on the overall 30-day postoperative morbidity of the procedures performed [7]. High risk colorectal procedures were defined as those having ≥ 35% morbidity rate for open procedures and ≥ 25% morbidity rate for MIS procedures (due to the lower overall morbidity associated with MIS colorectal procedures). High risk hepatic procedures were defined as those having ≥ 35% morbidity rate for open and MIS procedures. A simultaneous procedure was considered high risk if either the colorectal or hepatic resection were high risk.

The primary outcomes were procedure-specific 30-day postoperative complications including ileus and anastomotic leak for colorectal procedures and liver failure, bile leak, or need for a hepatic invasive procedure for hepatic procedures. Secondary outcomes included an unplanned conversion to open procedure and 30-day postoperative overall morbidity, serious morbidity, readmission, reoperation, mortality, and LOS. This study was approved by the Institutional Review Board of the Johns Hopkins School of Medicine.

## RESULTS

A total of 1,550 patients were identified who underwent a simultaneous resection. Of these patients, 311 (20%) underwent resection by an MIS approach, defined as an intended laparoscopic (n=241, 78%) or robotic procedure (n=70, 23%). A planned open approach was utilized in 1,239 (80%) patients. Patients who underwent robotic surgery were younger (51.5 vs. 62.0 years, p<0.001), had lower ASA class (p=0.042), and more frequently had received preoperative chemotherapy (71.9% vs. 55.9%, p=0.008) than patients who underwent laparoscopic surgery. The majority of MIS procedures were considered low risk (robotic: 91%, laparoscopic: 87%), compared to 75% of procedures in the open group.

Colorectal-specific outcomes were available for 616 patients (40%) while hepatectomy-specific outcomes were available for 875 patients (56%). Patients who underwent robotic resections had lower rates of ileus compared to the open group but similar rates to the laparoscopic group (Table 1). There were no differences in rates of 30-day anastomotic leak between any groups. No hepatectomy-specific complications were reported among patients who underwent a robotic procedure.

The rate of conversion to open procedure was lower for robotic compared to laparoscopic cases (9% vs. 22%, p=0.012). Rates of 30-day postoperative overall morbidity (26.6% vs. 27.1%, p=0.930), serious morbidity (7.8% vs. 15.4%, p=0.124), readmission (8.4% vs. 9.6%, p=0.963), reoperation (3.1% vs. 4.3%, p=0.999), mortality (0 vs. 3.1%, p=0.575), and median LOS (5 vs. 5, p=0.957) did not differ between the robotic and laparoscopic groups, respectively.

## DISCUSSION

A multidisciplinary approach with advances in comprehensive cancer care have led to more treatment options for patients presenting with resectable CRLM. Surgical resection is the preferred definitive treatment; however, resection in a simultaneous versus staged fashion remains controversial Although prior reports have utilized national databases to investigate the risk of simultaneous resections, this is the first report to 1) specifically investigate outcomes of robotic simultaneous resections, and 2) compare colorectal and hepatectomy-specific risks between open, laparoscopic, and robotic surgical groups. Our report found that although numbers of robotic resections were small (n=70), the rates of ileus, anastomotic leak, bile leak, hepatic failure, and need for invasive hepatic procedures post operatively were low, and rates of conversion to open were superior compared to laparoscopic cases.

Prior reports on MILS have been case series and small single-institutional studies. A systemic review of 9 studies investigating robotic assisted simultaneous resections for patients with synchronous CRC and CRLM included a total of 29 patients and found the overall rate of any morbidity was 38%, the rate of serious morbidity was 7%, and no perioperative deaths or conversion to open were noted [4]. Ten percent of patients experienced liver related complications, and only one patient experienced a colorectal complication (anastomotic leak); however, there was no comparison to rates from laparoscopic cases. In our cohort of 70 patients, rates of morbidity and complications were comparable, further adding to the literature on the safety of this approach in select patients with the potential benefit of decreased rates of conversion to open.

While this data suggest that the robotic platform can be safely utilized with potential benefits in patients with CRC and CRLM, cost and widespread feasibility are important considerations. Although studies report that MILS is on average less costly than open liver resections ($19,463 vs. $29,119), compared to laparoscopic surgery, robotic surgery has been shown to be more expensive in both isolated colorectal and hepatic procedures [8–12]. No study thus far has compared the costs of a robotic to laparoscopic approach in simultaneous CRC and CRLM resections. As more surgeons and centers expand their use of the robotic platform, future research on cost comparisons between the two MIS approaches is warranted.

This study is not without limitations. ACS-NSQIP is a national, standardized, multi-institutional database that focuses on the quality of surgical care but does not include hospital or surgeon specific variables. MILS is more likely to be performed at highly specialized centers, but due to limitations of the database, the authors cannot definitively comment on this. Despite utilizing a large national database, the numbers of patients who underwent a robotic simultaneous resection are still low. Lastly, patients were identified separately from either the hepatectomy or colectomy/proctectomy targeted files. As a result, information on procedure risk is limited to either the colorectal or hepatic procedure but not available for both.

The utilization of MIS approaches may mitigate some of the morbidity associated with simultaneous CRC and CRLM resections. As MILS becomes more common, patients may increasingly be offered a robotic approach to simultaneous resections. Data presented in this report suggests that robotic simultaneous resections can be performed without added procedure-specific risks. This information adds to the growing literature that the robotic platform can be used in increasingly complex procedures and may have additional benefits over a laparoscopic approach such as lower rates of conversion to open in simultaneous CRLM resections.

## Figures and Tables

**TABLE 1.** 30-Day Procedure-Specific Postoperative Complications from Procedure-Targeted NSQIP Files for Simultaneous Resections

	Colectomy/proctectomy-targeted NSQIP files			Hepatectomy-targeted NSQIP files			
Outcome (%)	**Open** 462 (37.3)	**Robotic** 50 (78.1)	**Lap** 104 (55.3)	**Open** 777 (62.7)	**Robotic** 14 (21.9)	**Lap** 84 (44.7)	***p*** (robotic vs lap)
Ileus^a^	114 (24.7)	5 (10.0)	20 (19.2)	-	-	-	0.146
Anastomotic leak^a^	17 (3.7)	2 (4.0)	4 (3.9)	-	-	-	0.999
Postop liver failure^b^	-	-	-	40 (5.2)	0 (0)	1 (1.2)	0.999
Postop bile leak^b^	-	-	-	44 (5.7)	0 (0)	1 (1.2)	0.999
Hepatic invasive procedure^b^	-	-	-	115 (14.9)	0 (0)	9 (10.7)	0.350

a Colectomy/proctectomy-specific outcomes available for patients with colectomy/proctectomy resection listed as a primary procedure in the NSQIP colectomy/proctectomy-targeted files only (n= 41%).

b Hepsatectomy-specific outcomes available for patients with hepatic resection listed as a primary procedure in the NSQIP hepatectomy-targeted files only (n=59%).

Abbreviations: NSQIP, National Surgical Quality Improvement Program; Lap, laparoscopic

## References

[1] Martin J., Petrillo A., Smyth E. C., Shaida N., Khwaja S., Cheow H., Duckworth A., Heister P., Praseedom R., Jah A., Balakrishnan A., Harper S., Liau S., Kosmoliaptsis V. & Huguet E. Colorectal liver metastases: Current management and future perspectives. World J. Clin. Oncol. 11, 761–808 (2020). 33200074 10.5306/wjco.v11.i10.761 PMC7643190

[2] Taylor A., Primrose, Langeberg W., Kelsh, Mowat F., Alexander D., Choti M., Poston G., & Gena Kanas. Survival after liver resection in metastatic colorectal cancer: review and meta-analysis of prognostic factors. Clin. Epidemiol. 283 (2012). doi:10.2147/CLEP.S34285 PMC3496330 23152705

[3] Snyder R. A., Hao S., Irish W., Zervos E. E., Tuttle-Newhall J. E. & Parikh A. A. Thirty-Day Morbidity after Simultaneous Resection of Colorectal Cancer and Colorectal Liver Metastasis: American College of Surgeons NSQIP Analysis. J. Am. Coll. Surg. 230, 617–627.e9 (2020). 32007534 10.1016/j.jamcollsurg.2019.12.018

[4] Machairas N., Dorovinis P., Kykalos S., Stamopoulos P., Schizas D., Zoe G., Terra A. & Nikiteas N. Simultaneous robotic-assisted resection of colorectal cancer and synchronous liver metastases: a systematic review. J. Robot. Surg. 15, 841–848 (2021). 33598830 10.1007/s11701-021-01213-8

[5] Driedger M. R., Yamashita T. S., Starlinger P., Mathis K. L., Smoot R. L., Cleary S. P. & Nagorney D. M. Synchronous resection of colorectal cancer primary and liver metastases: an outcomes analysis. HPB 23, 1277–1284 (2021). 33541806 10.1016/j.hpb.2021.01.002

[6] Moris D., Tsilimigras D. I., Machairas N., Merath K., Cerullo M., Hasemaki N., Prodromidou A., Cloyd J. M. & Pawlik T. M. Laparoscopic synchronous resection of colorectal cancer and liver metastases: A systematic review. J. Surg. Oncol. 119, 30–39 (2019). 30481373 10.1002/jso.25313

[7] Shubert C. R., Habermann E. B., Bergquist J. R., Thiels C. A., Thomsen K. M., Kremers W. K., Kendrick M. L., Cima R. R. & Nagorney D. M. A NSQIP Review of Major Morbidity and Mortality of Synchronous Liver Resection for Colorectal Metastasis Stratified by Extent of Liver Resection and Type of Colorectal Resection. J. Gastrointest. Surg. 19, 1982–1994 (2015). 26239515 10.1007/s11605-015-2895-z

[8] Miller A. T., Berian J. R., Rubin M., Hurst R. D., Fichera A. & Umanskiy K. Robotic-Assisted Proctectomy for Inflammatory Bowel Disease: A Case-Matched Comparison of Laparoscopic and Robotic Technique. J. Gastrointest. Surg. 16, 587–594 (2012). 21964583 10.1007/s11605-011-1692-6

[9] Wei D., Johnston S., Patkar A. & Buell J. F. Comparison of clinical and economic outcomes between minimally invasive liver resection and open liver resection: a propensity-score matched analysis. HPB 23, 785–794 (2021). 33046367 10.1016/j.hpb.2020.09.017

[10] Ramji K. M., Cleghorn M. C., Josse J. M., MacNeill A., O’Brien C., Urbach D. & Quereshy F. A. Comparison of clinical and economic outcomes between robotic, laparoscopic, and open rectal cancer surgery: early experience at a tertiary care center. Surg. Endosc. 30, 1337–1343 (2016). 26173546 10.1007/s00464-015-4390-8

[11] Baek S.-J., Kim S.-H., Cho J.-S., Shin J.-W. & Kim J. Robotic versus Conventional Laparoscopic Surgery for Rectal Cancer: A Cost Analysis from A Single Institute in Korea. World J. Surg. 36, 2722–2729 (2012). 22855217 10.1007/s00268-012-1728-4

[12] Khorgami Z., Li W. T., Jackson T. N., Howard C. A. & Sclabas G. M. The cost of robotics: an analysis of the added costs of robotic-assisted versus laparoscopic surgery using the National Inpatient Sample. Surg. Endosc. 33, 2217–2221 (2019). 30327915 10.1007/s00464-018-6507-3

